# Polypyrrole fabricated Laponite RD conductive nanocomposites: influence of grafting efficiency on structural and conductive properties

**DOI:** 10.55730/1300-0527.3617

**Published:** 2023-06-19

**Authors:** Shafiq Ur REHMAN, Shaista TAIMUR, Asif RAZA, Tariq YASIN

**Affiliations:** 1 Department of Chemistry, Faculty of Applied Sciences, Pakistan Institute of Engineering and Applied Sciences, Islamabad, Pakistan; 2 Department of Chemistry, University of Wah, Pakistan

**Keywords:** Laponite RD, polypyrrole, emulsion polymerization, conductive nanocomposite

## Abstract

In the present work novel conductive organic-inorganic nanocomposites were produced by grafting of pyrrole monomer onto silanized Laponite RD utilizing emulsion graft polymerization. Influence of some important factors like concentration of monomer, initiator and surfactant were investigated on grafting efficiency. Grafting of polypyrrole chains onto modified Laponite RD was verified by Fourier transform infrared spectroscopy (FT-IR). Scanning electron microscope (SEM) revealed the spherical particles of nanocomposite with average diameter of 271.5 nm. XRD pattern showed that molecular framework of pure polypyrrole almost remains same in nanocomposite. Surface area and pore volume of Laponite RD, measured by Brunauer-Emmett-Teller (BET) analysis, was also altered indicating effective grafting of polypyrrole chains onto modified substrate. Maximum grafting efficiency (%), determined gravimetrically, was 87.3% at monomer, initiator, and surfactant concentrations of 1.50, 1.00, and 0.50% respectively. Prepared nanocomposites with grafting efficiency of 87.3% have displayed maximum electrical conductivity of 0.23 × 10^−2^ Scm^−1^. These nanocomposites can be used for manifold applications like biomedical and energy storage devices.

## 1. Introduction

Clay polymer nanocomposites (CPNs) are receiving great response by engineering minds due to their synergistic properties and widespread applications in medical field [1–7], environmental remediation [8–11] and sensor technology [12–14], etc. Different strategies have been adopted to prepare nanocomposite materials e.g., in situ polymerization (emulsion and radiation induced graft polymerization), solution blending, melt extrusion, spray drying [15–18], etc. Among these, emulsion graft polymerization is more suitable because tailored properties can be achieved by varying the reaction parameters. Moreover, use of aqueous solvent makes this technique a greener approach and a source of heat dissipation. Viscosity remains same during the course of reaction because polymerization takes place within the micelles [19].

High surface area, high aspect ratio and easy functionalization of clay to tackle hydrophilic nature of clays make them suitable to act as a base material in CPNs. Laponite RD, a 2:1 type synthetic hectorite clay with chemical formula Si_8_Mg_5.45_Li_0.4_O_20_OH_4_Na_0.7_, has surface area, aspect ratio and density of 370 m^2^/g, 25:1 and 2.6 g/cm^3^, respectively [20–22]. It is phyllosilicate clay, and each disk is made of central octahedral magnesium oxide-hydroxide layer sandwiched between two SiO4 tetrahedral layers. These disks are separated from one another by exchangeable interlayer cations (Na^+^); therefore, term sodic-Laponite (Na-L) is most commonly used [23]. Periodic upside-down inversion of tetrahedral sheets exposes the Si-OH (4 μm/m^2^) group on edges [21]. Thus surface modification of Laponite by several coupling agents is favorable due to exposed Si-OH groups [24–26]. Several coupling agents are used for surface modification of Laponite [24,27–36].

Among most common inherently conductive polymers, polypyrrole (ppy) is of considerable attention. Due to light weight and environmental stability [37], they may be used in electronics and electrical field as new material such as electromagnetic shielding [38], semiconductors [39], sensors [40], energy storage devices [41,42], etc. Their properties can be manipulated by making their nanocomposite.

The main focus of the present work is to prepare novel and efficient conductive polypyrrole/Laponite RD (ppy/Lap) nanocomposite. Novelty lies in the synthesis of polymer nanocomposite by grafting polypyrrole onto inexpensive Laponite nanoclay by a greener approach: emulsion graft polymerization. Vinyl functionalization of Laponite RD is done by vinyl triethoxysilane to graft pyrrole on it. Organic phase of nanocomposite material is grafted on base material by in situ polymerization of desired monomer. To achieve maximum degree of grafting, concentration of monomer, water soluble initiator and surfactant were optimized. Structural and morphological changes were also investigated in relation to the degree of grafting. Electrical conductivity is also explored to discover the potential of the developed nanocomposites as conducting CPNs. This study is intended to develop a promising candidate for those applications which require efficient conducting polymer nanocomposites such as biomedical, sensors, EMI (electromagnetic interference) shielding and ESD (electrostatic discharge) materials etc.

## 2. Experimental

### 2.1. Materials and chemicals

Laponite RD was purchased from Rockwood Additives (Cheshire, UK). Pyrrole monomer (98%), vinyl triethoxysilane (VTES, 97%), toluene (≥ 99.5%), hydrochloric acid (37%), acetone (≥ 99.5%), tetrahydrofuran (≥ 99.5%), ethanol (96%), and tween 80 (TW80, low carbonyls and low peroxides) were purchased from Sigma-Aldrich, Germany. Potassium persulfate (KPS, 98%) purchased from Daejung Chemicals, Korea was used in this study.

### 2.2. Synthesis

#### 2.2.1. Organic modification of Laponite RD

Laponite RD was washed by dispersing 10 g of Laponite in 1 L of ethanol. After 24 h, solution was filtered, and residue was dried in vacuum at 60°C until constant weight. Acid activation of Laponite RD was done according to the reported method [43]. Briefly 1 g Laponite RD was dispersed in distilled water (150 mL) at 1250 rpm. After 30 min, 0.1 M HCl solution (100 mL) was added to the dispersion and again stirred. After 3 h, solution was filtered and washed with distilled water to remove in situ formed NaCl. Acid activated Laponite (AL) was now dried in a vacuum oven at 60°C until constant weight and then stored in desiccator for further use.

**Figure 1:**
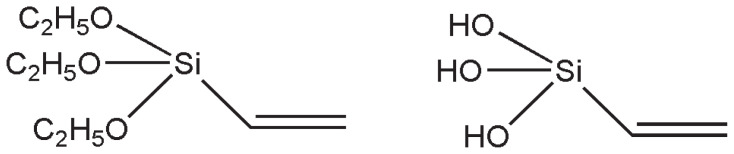
VTES and hydrolyzed VTES.

Vinyl functionalization of Laponite RD was done by the reported method [29]. AL (1 g) was mechanically dispersed at 1250 rpm in 10 mL of toluene in a glass reactor equipped with condenser followed by dropwise addition of 10.44 mmol of hydrolyzed VTES [18,44]. VTES was hydrolyzed by the addition of 2–3 drops of 1% HCl and their structures are shown in Figure 1.

After 72 h, product was filtered and washed with toluene. Vinyl modified Laponite (ML) was dried in vacuum oven at 60 °C until constant weight.

#### 2.2.2. Emulsion polymerization

In a glass reactor fixed with condenser on a water bath, 0.1 g ML and TW80 (0.75 g) were mechanically dispersed in 100 mL of 1 M HCl for 20 min. Stirring was done in an inert atmosphere. Pyrrole monomer (2 g) was introduced into this reactor slowly followed by the dropwise addition of 2 g initiator (KPS) dissolved in 100 mL of 1M HCl solution. Reaction mixture was heated up to 70°C. After 8 h, black product was filtered and washed with demineralized water, acetone, and tetrahydrofuran, respectively, to remove homopolymer. The polypyrrole grafted Laponite (ML-g-ppy) was dried in vacuum oven at 60°C until constant weight. Grafting percentage and grafting efficiency (GE) was calculated according to the following formulae:


$$\begin{array}{r} \text{Grafting }\%=\frac{{\text{W}}_{\text{g}}-{\text{W}}_{\text{i}}}{{\text{W}}_{\text{i}}}\times 100\\ \text{GE }(\%)=\frac{W_{g}-W_{i}}{W_{i}}\times 100, \end{array}$$

where W_g_, W_i_, and W_m_ are the weights of ML after grafting of pyrrole monomer, ML initially taken and pyrrole monomer respectively.

### 2.3. Characterization

#### 2.3.1. FTIR

Thermo Scientific, USA, Nicolet 6700 infrared spectrophotometer was used to record FTIR spectra of nanohybrid material. Fourier transform infrared spectrophotometer- attenuated total reflection (ATR) technique with diamond crystal was used. Scanning range of 4000–400 cm^−1^ was used for all materials. High signal to noise ratio was obtained by recording the 200 average scans per sample at resolution of 6 cm^−1^.

#### 2.3.2. SEM and EDX

Surface morphology was studied by using Tescan MIRA–3 field emission scanning electron microscope. The sputtering of the sample was done using CCU-010 compact coating unit. At different magnifications, images were analyzed. The said instrument was coupled with EDX for elemental analysis.

#### 2.3.3. X-ray diffraction

D8 Discover X-ray diffractometer with nickel-filtered Cu Kα radiation (λ¼ 1.542 Å) at 30 mA was used to record XRD of nanohybrid material. Scanning angle was from 3° to 30°.

#### 2.3.4. BET analysis

ASAP 2020 Analyzer was used for BET analysis of pristine Laponite RD (PL), ML, and composite material using liquid nitrogen. All samples were purified by degassing and vacuum dried before analysis. The amount of nitrogen gas adsorbed is directly related to the surface area of the sample.

#### 2.3.5. Electrical conductivity

Electrical conductivity was determined by making compressed pellets without adding any binder (diameter 10 mm and thickness ≈ 1 mm) at room temperature and the Ossila Four-Point Probe System was used for DC conductivity determination.

#### 2.3.6. Statistical analysis

All experiments were run in triplicate and results were computed with standard deviation of ± 2.

## 3. Results and discussion

### 3.1. Synthesis of ppy/Lap nanocomposite

**Figure 2:**
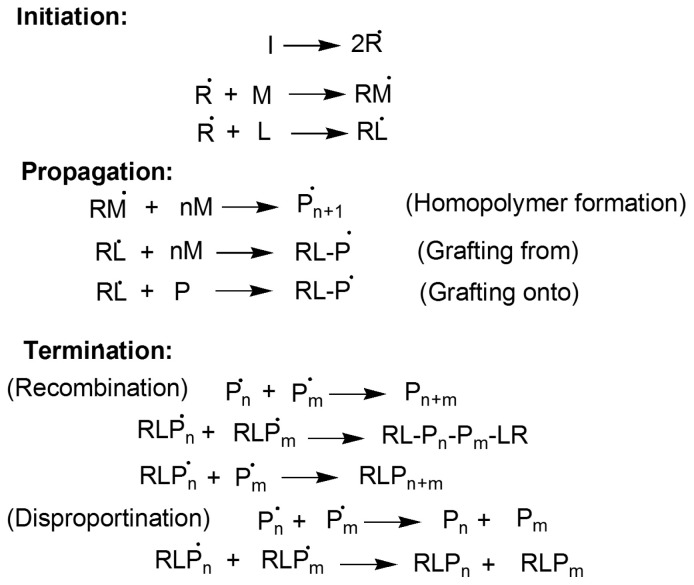
Sequence of possible reactions during grafting.

**Figure 3:**
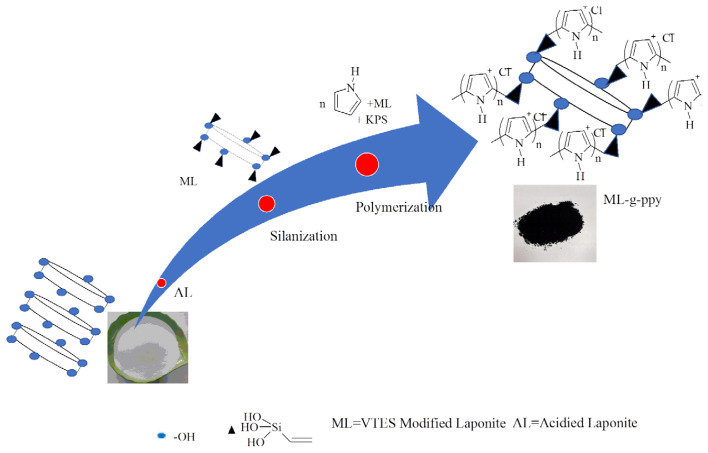
An overview of all steps involved in synthesizing ML-g-ppy.

Free radicals initiate polymerization which were generated by thermal decomposition of initiator, KPS. These radicals enter the micelles and interact with ML, and pyrrole monomer already present within micelles to initiate polymerization. Free radicals produced by monomer (RM.) and (RL.) further react with pyrrole monomer to generate polymer chains. The sequence of possible reactions during grafting is shown in Figure 2 whereas the whole process of nanocomposite development is capsuled in Figure 3.

Grafting on base material may take place either by “grafting onto” or “grafting from” approach. Polymerization is terminated either by recombination of polymeric chains bearing radical or by disproportionation reaction i.e. by hydrogen transformation.

### 3.2. Optimization of conditions for the preparation of ppy/Lap nanocomposite

Number of parameters such as concentration of surfactant, monomer and initiator can affect the grafting process. Therefore, these parameters are required to be optimized to achieve maximum grafting of ppy on Laponite RD.

#### 3.2.1. Optimization of monomer concentration

**Figure 4:**
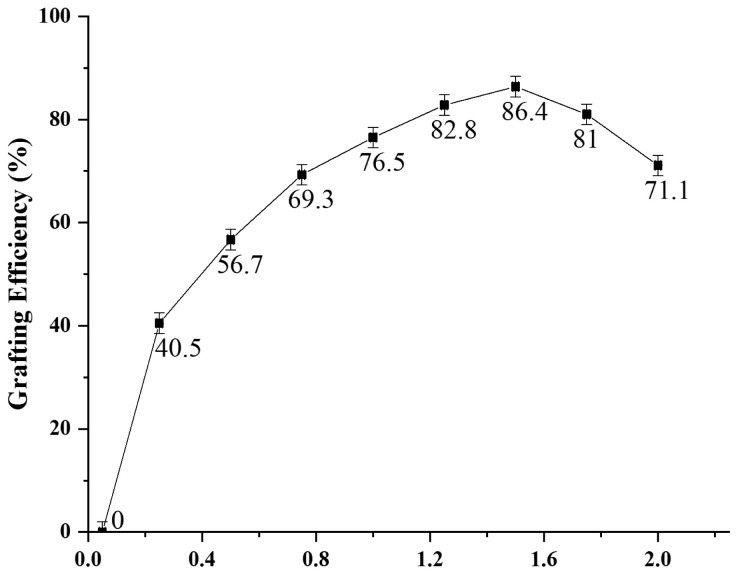
Effect of monomer variation on grafting of pyrrole on Laponite RD.

While keeping initiator and surfactant concentration fixed, monomer concentration was varied from 0.05% to 2.00% and results are shown in Figure 4. It is clear from the graph that GE (%) increases with increasing the monomer quantity up to certain level. At very low concentration of monomer, minimum GE was observed that means number of monomer molecules were insufficient for grafting onto Laponite RD that contains enough number of grafting sites.

After certain concentration of monomer, GE decreases that means all grafting sites of Laponite RD were already occupied and homopolymer is formed which lessens the accessibility of pyrrole monomer to ML. This means that GE not only depends upon the number of free radicals formed but is also dependent on the diffusion of pyrrole monomer to ML. Maximum GE was obtained at 1.50% concentration of monomer. Results were comparable to the works done by Raza et al. (2020) and Taimur et al. (2018) [8,16,44–46].

#### 3.2.2. Optimization of initiator concentration

**Figure 5:**
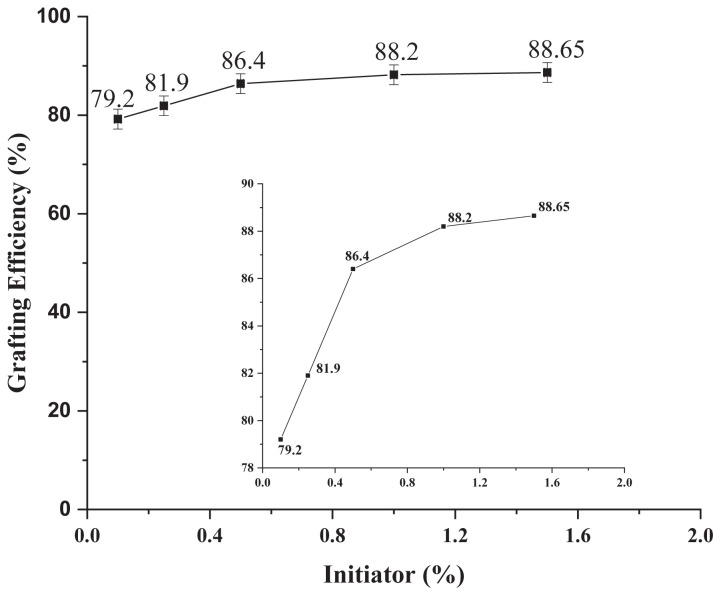
Effect of initiator variation on grafting of pyrrole on Laponite RD.

Initiator was varied while keeping the monomer and surfactant variation constant and results are shown in Figure 5 and are in agreement to the work done by Raza et al. (2020) [16,45].

GE increases linearly with increasing the initiator concentration. This may be due to increase in number of free radicles both on base material and pyrrole monomer, thus increasing crosslinking and grafting. As GE is almost same at 1.0 and 1.5% of initiator, thus for cost economy, 1.0% optimized condition was used in successive experiments.

#### 3.2.3. Optimization of surfactant concentration

**Figure 6:**
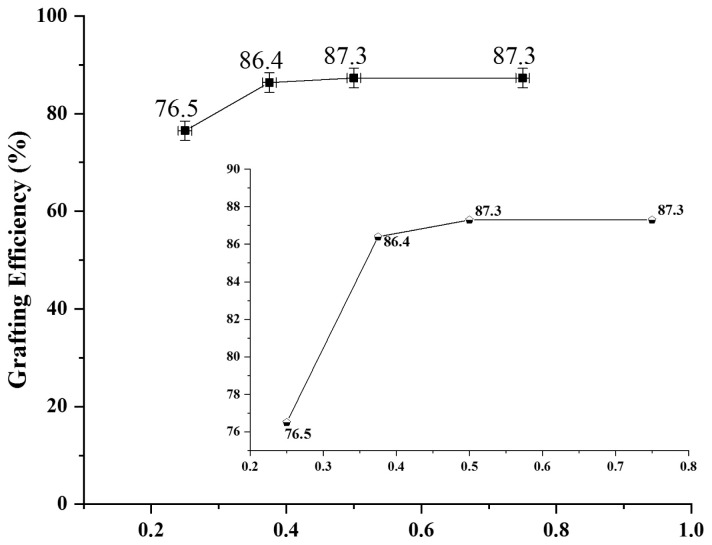
Effect of surfactant variation on grafting of pyrrole on Laponite RD.

In emulsion polymerization, surfactant plays an important role in forming micelles and stable suspension of Laponite RD and monomer molecules. Polymerization takes place in these micelles. The effect of surfactant was studied using optimized concentrations of monomer and initiator and results are depicted in Figure 6.

GE increases with increasing the surfactant concentration up to a maximum value then almost becomes constant because at that point, there are enough micelles for the polymerization to be carried out. Results obtained were in agreement with the cited literature [16,45].

### 3.3. Characterization

#### 3.3.1. FTIR study

**Figure 7:**
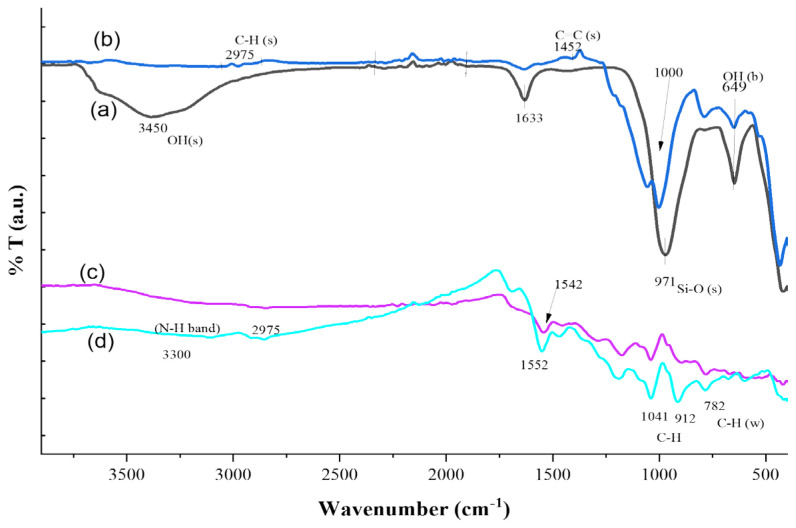
FTIR spectra of (a) PL, (b) ML, (c) ppy, and (d) ppy/Lap nanocomposites.

FTIR spectra of PL, ML, ppy, and ppy/Lap nanocomposite are shown in Figure 7. Spectrum (a) represents the PL, and the most important diagnostic signals are: OH bending vibration of Mg-OH at 649 cm^−1^ and Si-O stretching vibration band was observed at 971 cm^−1^. These vibrations were comparable to the works of Madejova (2003) and Palkova et al. (2009) [47,48]. Position change of this band after silane incorporation into Laponite RD confirms structural changes in PL as evident from spectrum (b). Zeolitic water molecules show peak at 1633 cm^−1^. OH stretching vibration band was observed centered around 3450 cm^−1^.

Spectrum (b) represents the ML where stretching vibration at 1452 cm^−1^ is attributed to starching of C=C of pyrrole ring [17]. In this spectrum, symmetric stretching vibration of =CH2 at 2975 cm^−1^ was also observed. Spectrum (c) represents ppy and peaks at 912 and 1041 cm^−1^ are due to C-H out of plane vibrations and C-H in plane vibrations and these results are comparable to the work of Khadem et al. (2017)[49]. Peak at 782 cm^−1^ is due to C-H wagging vibrations. Peak at 1542 cm^−1^ corresponds to C=C and C-C stretching vibrations. C-C bonds of pyrrole rings show stretching vibrations at 1452 cm^−1^. This region also confirms the presence of conjugation in ppy. The shoulder observed at 1284 cm^−1^ corresponds to in plane deformation of C-H and C-N bonds. Large band around 3300 cm^−1^ is due to N-H symmetric stretching vibration. Grafting of ppy on base material is shown in spectrum (d) and this shows the characteristic peaks of both Laponite as well as ppy. Decrease in Si-O stretching vibration may be due to utilization of this group during polymerization. These results are comparable to the results reported by Fu et al. (2012) [50]. With the aid of FTIR, we can conclude that spheres observed in SEM micrographs belong to ppy/Lap nanocomposite.

#### 3.3.2. Scanning electron microscopy

**Figure 8:**
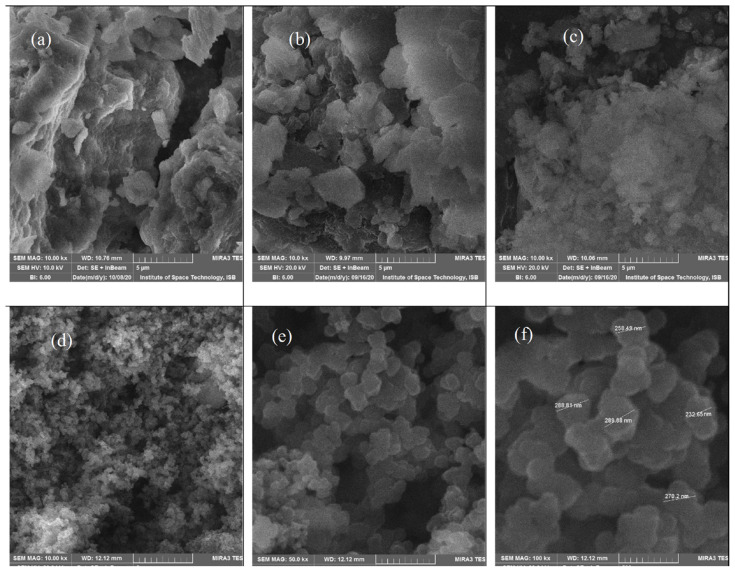
SEM images of (a) PL, (b) AL, (c) ML, (d, e, and f) ppy/Laponite composite at 5 μm, 1 μm, and 500 nm resolution.

SEM is capable of resolving particle size and shows surface morphology of synthesized particles. SEM images (Figure 8) of Laponite RD and synthesized material are shown by placing the samples at 5 μm, 1 μm, and 500 nm. As agglomeration of clay particles has taken place, laminar structure is not observed in SEM micrographs (Figure 8a). By treatment with acid (Figure 8b), these sheets are exfoliated as manufacturer has reported [51]. Similar results were also reported by Mejia et al. (2017). Crystalline nature of Laponite was not observed in ML (Figure 8c) as also revealed by XRD analysis and peak shifting in FTIR.

In ppy/Lap nanocomposite (Figures 8d–8f), spherical particles having average particle diameter of 271.3 nm were observed. These results are comparable to the works reported by Sevil and Zuhal (2010) and Khadem et al. (2017) [49,52]. These micrographs show the amorphous nature of agglomerated ppy/Lap RD nanocomposites which can be verified by Ahmad et al. (2016) [12] and is also noticeable and verified from XRD patterns of these microspheres. No clear boundary around these spheres may be due to crosslinking of polymeric chains [53].

#### 3.3.3. Energy dispersive X-ray spectroscopy (EDX)

**Figure 9:**
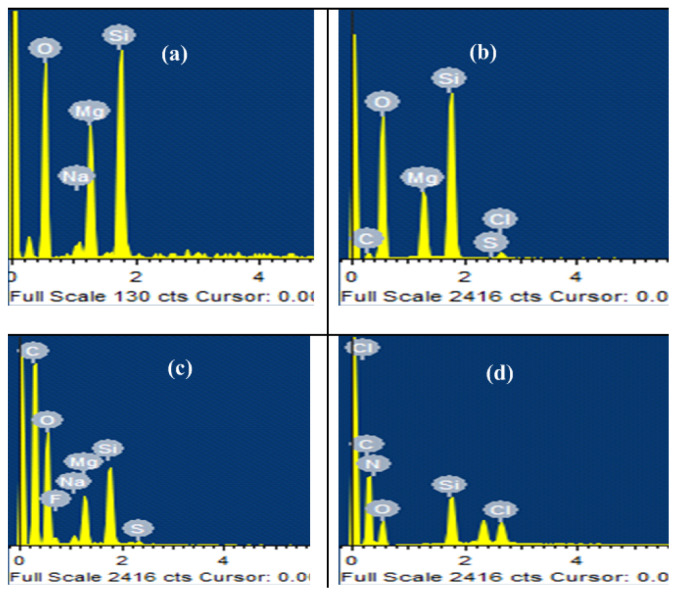
EDX Elemental analysis of (a) PL, (b) AL, (c) ML, and (d) ppy/Lap nanocomposite.

EDX is a semiquantitative analysis and EDX patterns and elemental composition of PL, AL, ML and ppy/Lap nanocomposite are shown in Figures 9a–9d and Table 1, respectively. Laponite RD has chemical formula Si_8_Mg_5.45_Li_0.4_O_20_OH_4_Na_0.7._ Therefore, Na, Mg, O, and Si are detected by EDX elemental analysis as also reported by Ituah (2012)[54].

Li is not detected in EDX analysis because of its lower atomic number. Increase in carbon contents after VTES modification confirms grafting of VTES on Laponite.

Appearance of Si in ppy/Lap nanocomposite confirms that grafting of ppy on clay has taken place. Detection of nitrogen and chlorine in composite confirms formation of chlorine doped ppy/Lap nanocomposite.

#### 3.3.4. XRD analysis

**Figure 10:**
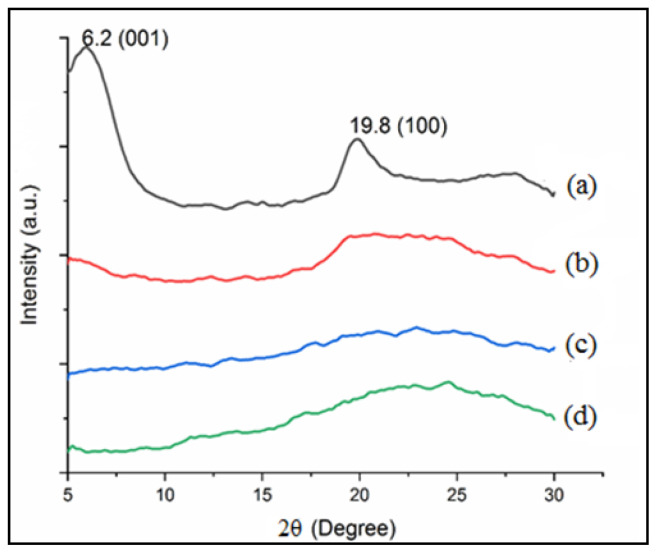
XRD diffractogram of (a) PL, (b) ML, (c) ppy, and (d) ppy/Lap nanocomposite.

XRD is a powerful tool to check the crystallinity of a material, polymer chains structure and their spatial order in space. XRD patterns of PL, ML, ppy, and ppy/Lap are presented in Figure 10.

PL is crystalline in nature as evident from diffractogram (Figure 10a). Reflections of PL were observed at 2θ = 6.2° (001) with a shoulder around 5.8°, and 2θ = 19.8° (100) corresponding to basal plane spacing of 14.2 Å and 4.47 Å, respectively. After VTES modification, crystalline nature of Laponite RD is diminished due to grafting of silane as evident from diffractogram of ML (Figure 10b). None of PL reflections were observed in ML diffractogram indicating that complete exfoliation of clay has taken place and VTES interaction with individual clay layer surface has taken place. These results are similar to the reported literature [30,55,56]. XRD patterns of Laponite RD based composite are difficult to obtain because of low crystallinity and exfoliation of the base materials as also discussed by Pereira et al. (2007) [29]. Grafting of ppy on individual clay layer surface has taken place resulting a completely exfoliated amorphous nanocomposite as evident from diffractogram of ppy/Lap (Figures 10c and 10d). Common counter ions e.g., Cl- doped ppy mostly exhibit amorphous structure because during oxidative polymerization, mostly branching or crosslinking takes place. This is in agreement with SEM micrographs. Most of the ppy composites give broad XRD pattern around 2θ =18–27° as cited in the literature [14,57–59].

#### 3.3.5. BET analysis

Surface area (m^2^/g) and pore volume (cm^3^/g) was calculated by BET technique. PL has large surface area of around 350 m^2^/g because of small particle size [11,60].

Surface area of Laponite decreases by attachment of VTES group. Surface area further decreases after the grafting of ppy because polymer chains fill the empty spaces which are formed because of house of cards structure in Laponite RD [45]. These results are presented in Table 2.

#### 3.3.6. Electrical conductivity

The electrical conductivity of ppy/Lap composite having different GE was measured, and results are comparatively shown in Table 3.

Electrical conductivity increased to 0.23 × 10^−2^ S/cm at GE of 87.3%. This may be due to increase in intercalation of polymer chains between the interlamellar spaces of Laponite [45,61] and formation of nanostructure between clay and polymer providing efficient pathway for charge transfer [41].

## 4. Conclusion

A novel conductive ppy/Lap nanocomposite material was successfully synthesized by a greener approach: emulsion polymerization. Maximum GE of 87.3% was obtained under optimized condition of monomer (1.50%), initiator (1.00%) and surfactant (0.50%). Structural changes in Laponite RD after vinyl functionalization and grafting were confirmed by FTIR and XRD. Morphological changes in Laponite RD during surface treatments were observed by SEM images showing conversion from crystalline structure to amorphous one. Appearance of carbon content after vinyl functionalization of Laponite confirmed the surface modification of Laponite as clear from EDX elemental analysis. Appearance of Si and Cl in nanocomposite confirmed the grafting of ppy chains on Laponite RD and Cl doping of composite material respectively as verified by EDX. ppy/Lap nanocomposite has presented its conductive nature with electrical conductivity of 0.23 × 10^−2^ S/cm (87.3% GE). The possibility of using prepared nanocomposite as EMI shielding material is also in progress.

## Figures and Tables

**Table 1:** Elemental compositions of different samples measured by EDX.

Element	PL (atomic %)	AL (atomic %)	ML (atomic %)	ppy/Lap (atomic%)
O	69.30	63.03	32.79	22.75
Na	1.48	-	0.45	-
Mg	11.55	6.77	1.88	-
Si	17.67	14.10	2.40	2.79
C	-	-	4.3	60.95
N	-	-	-	12.03
Cl	-	0.55	-	1.48

**Table 2:** Surface area and pore volume measured by BET technique.

Sample	Surface area (m^2^/g)	Pore volume (cm^3^/g)
ML	338	11.3
ppy/Lap composite GE = 45 %	136.2	2.6
ppy/Lap composite GE = 87.3 %	55.9	0.1

**Table 3:** DC conductivity of ppy/Lap and other composites.

Material	Grafting percentage (%)	Electrical conductivity (S/cm)	References
ppy/Lap composite	45	0.34 × 10^−^^4^	This study
ppy/Lap composite	87.3	0.23 × 10^−^^2^	This study
MS-g-ppy	-	0.85 × 10^−^^4^	[45]
ppy-Al-PMMT	-	05 × 10^−^^4^	[61]
1 PM/Fe	-	49.1	[62]
ppy/MMT	-	Range of 10^−^^4^ and 10^−^^3^	[42]
MMT/ppy	-	4.81	[63]
ppy/Mag-CTA + (2 CEC)	-	1.41	[40]
